# Comparison of Canal Transportation, Separation Rate, and Preparation Time between One Shape and Neoniti (Neolix): An In Vitro CBCT Study

**DOI:** 10.1155/2021/6457071

**Published:** 2021-09-07

**Authors:** Maryam Kuzekanani, Faranak Sadeghi, Nima Hatami, Maryam Rad, Mansoureh Darijani, Laurence James Walsh

**Affiliations:** ^1^Endodontology Research Center, Department of Endodontics, Kerman Dental School, Kerman University of Medical Sciences, Kerman, Iran; ^2^Student Research Committee, School of Dentistry, Kerman University of Medical Sciences & Health Services, Kerman, Iran; ^3^Oral and Dental Diseases Research Center and Kerman Social Determinants on Oral Health Research Center, Kerman University of Medical Sciences, Kerman, Iran; ^4^Department of Oral & Maxillofacial Radiology, School of Dentistry, Kerman University of Medical Sciences & Health Services, Kerman, Iran; ^5^UQ Oral Health Centre, The University of Queensland School of Dentistry, 288 Herston Road, Herston 4006, Australia

## Abstract

**Purpose:**

This in vitro study compared root canal preparation in curved mesiobuccal canals of molar teeth using either the One Shape™ or the Neoniti (Neolix) rotary NiTi single-file systems, assessing canal transportation, instrument separation and time required for preparation*. Methods*. Extracted maxillary and mandibular human molar teeth with mesiobuccal canals having apical angles of curvature between 25 and 35^o^ were selected and embedded in acrylic resin blocks, and an initial CBCT was taken. The teeth were divided into two equal groups (*n* = 20), and the canals were cleaned and shaped using either Neoniti™ or One Shape™ engine-driven NiTi rotary files. Each individual instrument was used to prepare 5 canals. The time required for the preparation of each canal was recorded. Postpreparation CBCT scans were taken and used to determine the extent of canal transportation at levels of 2, 4, 6, and 8 mm from the apex. The Kolmogorov–Smirnov test for normality was applied, and then, datasets were compared using independent *t*-tests, with a threshold of *P* < 0.05.

**Results:**

Neoniti rotary files caused significantly less canal transportation of the curved canals (*P*=0.0001). On the other hand, the time required for canal preparation was significantly shorter for One Shape (*P*=0.0001). No instrument separation was recorded in both groups.

**Conclusion:**

Based on these results, the Neoniti™ rotary file system is preferred because it maintains the original shape of curved root canals better than One Shape™; even though this benefit comes at the cost of an increase in preparation time in clinical practice, the better technical performance may be more important than a difference in procedural time.

## 1. Background

Development of nickel-titanium (NiTi) rotary instruments has improved the overall quality of canal preparation in endodontics, with fewer procedural errors, such as ledges, zipping perforations, and transportation [1]. Third- and fourth-generation files include Neoniti™ (Neolix, Chatres-La-Foret, France) and One Shape™ (Micro-Mega, Besançon, France), respectively. These both employ a single file used in continuous rotating motion to clean and shape the whole root canal system [2]. The main advantages of such single rotary NiTi file systems are ease of use and high efficiency, reducing the time required, which benefits both the clinician and the patient.

Neoniti™ files are available in three different sizes (20/0.08, 25/0.08, and 40/0.08). According to the manufacturer, they should be used at a rotational speed of 300 to 500 rpm and a torque limit of 1.5 N/cm, for single-length preparation to the working length [3]. The files are manufactured using a wire-cut electrical discharge machining (WEDM) process. This gives the files a rough surface, with abrasive properties, to facilitate root canal preparation. Heat treatment of these files during their production enhances their flexibility [4–6]. On the other hand, One Shape™ files have a variable pitch, with a safe noncutting tip, and three different cross-sectional profiles along the active length: a triangular or modified triangular cross section and three sharp cutting edges in the middle and apical thirds and an S-shaped cross section with two cutting edges near the shaft [7].

Using files in severely curved canals is challenging because the canal anatomy increases the likelihood of transportation, with the file not conforming exactly to the curved profile of the canal despite its flexibility [8]. Therefore, an evaluation of the extent of canal transportation is an important aspect of assessing a NiTi rotary file system. This laboratory study aimed to compare canal transportation, preparation time, and instrument separation between One Shape and Neoniti when used to prepare severely curved root canals. The null hypothesis was that there was no difference between the two systems in terms of these parameters.

## 2. Material and Methods

A total of 40 human mandibular and maxillary molar teeth extracted because of periodontal diseases or prosthetic treatments were collected, with the approval of the institutional ethics committee. All selected teeth had at least one curved and operable mesiobuccal canal. The initial file in all selected mesiobuccal canals was a no. 15 K file, so the selected canals had similar diameters. The crown of each tooth was removed at the level of the cementoenamel junction with a diamond bur, to obtain a mesiobuccal root canal, with a root length of approximately 12 mm. The teeth were embedded into acrylic resin, and then radiographed with an ISO 15 K file in the curved mandibular or maxillary mesiobuccal canals [7–10]. The degree and radius of root canal curvature were determined from the radiographs using the Schneider and the Pruette analysis method [11, 12]. Only roots with angles of curvature between 25 and 35° and radii of curvature between four and nine were used in the study. The selected samples were then divided randomly into two experimental groups of 20 teeth each.

### 2.1. Sample Size Calculation Method

The sample size was calculated for  = 0.05. using the following parameters:

- *P* = 0.9
- *M*1 = 1.8
- *M*2 = 2.8
- SD1 = 0052
- SD2 = 0.4

using the STATA program. This gave 18 or 20 in each group.

STATA program: n1 = n2 = 18 or 20 in each group.

In one group, the canals were prepared using Neoniti, following the manufacturer's instructions. A glide path was established in each canal, using a stainless steel #15 K-file (Dentsply Maillefer) to the working length. Root canal preparation was performed by an endodontic motor (Endo IT, NSK, Kanuma, Japan). Canals were instrumented by a C1 file in the coronal third, followed by an A1 file (#25/0.08) with in-and-out motion in the middle and apical thirds, at 300 to 500 rpm and with a torque limit of 1.5 N/cm.

In the other group, the same general approach was followed, first establishing a glide path with a #15 K-file (Dentsply Maillefer) to the working length and then a One Shape #25/0.06 file to two thirds of the working length to clean and shape the curved canal, again using an in-and-out motion. Then, a #10 K file was used to check the working length, and this was followed by the One Shape #25/0.06 file, this time to the full working length, to enlarge and shape the apical third.

With both file systems, copious irrigation was performed during instrumentation, using 2.5% NaOCl and alternating this with 17% EDTA. Each file was used to prepare only 5 root canals [3–5, 8–10]. The time required for the preparation was recorded, as was any occurrence of instrument separation.

**Figure 1:**
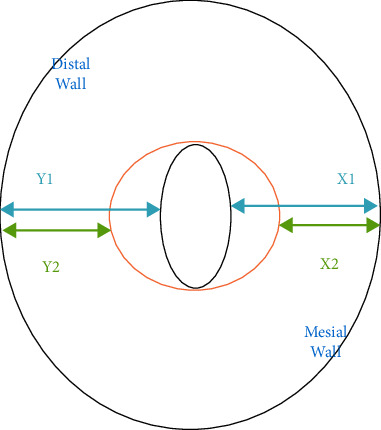
Schematic photo which explains the formula used for the transportation assessment.

**Figure 2:**
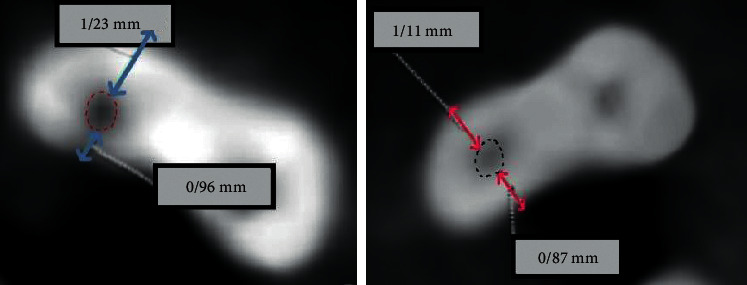
Image of a sample showing the location of X1, X2, Y1, and Y2.

**Figure 3:**
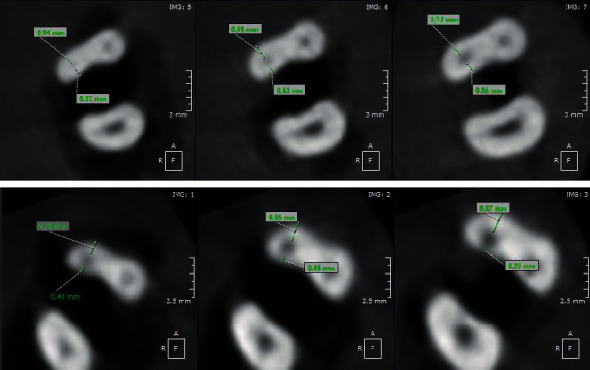
Typical CBCT images of samples.

Before and again after instrumentation, CBCT scans were taken from the roots, using a dental CBCT system (Pax-i3D version 1.0.0.7, VaTeCH, South Korea) with the following parameters: 110 kVp, 9.5 mA, 12 sec, 0.1 × 0.1 x 0.1 voxel sizes, and 0.100 mm axial thickness. CBCT data were processed using OnDemand 3D digital imaging software (version 1.0.10.53850). In each dataset, first the root tip location was designated, and then cross-sectional horizontal slices were taken from the mesiobuccal canal at four locations: 2, 4, 6, and 8 mms coronally from the apex. Measurements from the pre- and postinstrumentation scans at the four locations were made, to the nearest 0.1 mm. The extent of canal transportation was calculated using this formula: (Y1–Y2)−(X1–X2), where *Y*1 = the shortest distance between the canal's distal wall and the root's distal periphery before instrumentation; *Y*2 = the shortest distance between the canal's distal wall and the root's distal periphery after instrumentation; *X*1 = the shortest distance between the canal's mesial wall and the root's mesial periphery before instrumentation; and *X*2 = the shortest distance between the canal's mesial walls and the root's mesial periphery after instrumentation (Figures 1–3) [13–16]. For statistical analysis, the Kolmogorov–Smirnov test was applied to verify that all datasets showed a Gaussian distribution. Due to the normal distribution of datasets, independent *t*-tests were used to compare the two groups. The threshold for significance was set at *P* < 0.05.

## 3. Results

t-test statistical analysis between two groups showed that Neoniti rotary system gave a significantly less canal transportation and as a result was preferable for preserving the original shape of the curved canals (*P*=0.0001), no instrument from 2 brands separated through this study, and the preparation time in the One Shape group was significantly less than that in the Neoniti (*P*=0.0001).

Results of this study are summarized in Tables 1–3.

## 4. Discussion

Cleaning and shaping the root canal system is one of the most important phases in endodontic treatment. Safe, quick, and minimum change in the original shape of the prepared root canals are the gold standards expected from an efficient method for the purpose of root canal preparation [17]. CBCT is one of the most informative technologies for diagnosis and research in endodontics and has been shown to provide better results to assess the root canal change in the shape and transportation, than other methods such as the pre- and the postperiapical radiographies [18]. Although the gold standard for evaluation the shaping ability of the NiTi rotary instruments is micro-CT, CBCT has also provided valuable results in many studies by 3D scans from before and after root canal preparation [3, 6, 14, 15, 17, 19].

Manual Glide path was established in both experimental groups by a stainless steel #15 K-file (Dentsply Maillefer) to the working length in order to reduce torsional and threading in effects of the NiTi rotary files that cause instrument separation and the risk of canal transportations [16]. This is the first study that compares the properties of (Neoniti, Neolix, Chatres-la-Foret, France) with One Shape (Micro-Mega. France) both categorized in single-file NiTi rotary instruments, although there are many studies in the literature that have compared the shaping ability, fracture resistance, and speed of one of these single-file Niti rotary systems with other Niti rotary systems. Data presented in the Tables 1 and 2 show that significantly less canal transportation happens by the Neoniti system than the One Shape at all 4 distances from the root apex, and this difference is more evident in the apical one third (2 and 4 mms from the root apex) that decreases towards the coronal parts(6 and 8 mms from the root apex) that shows higher flexibility and tendency to straightening by the Neoniti instruments in apical regions of the curved root canals in comparison with the One Shape.

Moazzami et al. in a study in 2016 reported less canal transportion for Neoniti-Neolix than the reciproc(VDW) NiTi rotary system for preparation the curved root canals. In their study, just one Neoniti file separated [6]. In another study by Madani et al., there was no significant statistical difference in the amount of canal transportation between Neoniti and the protaper Niti rotary instruments [3].

Furthermore, in a study in 2017 (Neoniti, Neolix, Chatres-la-Foret, France), the single-file system showed less canal deviation and also needed less preparation time than the protaper universal system in curved root canals [4].

The results of current study are consistent with all past studies that have reported the shaping ability property of the Neoniti single-file system the same or better than other NiTi rotary systems under study. Less canal transportation by Neoniti may be due to its nonhomothetic rectangular cross section along with rounded Gothic tips [19]. Also, the Neoniti system does not have the tendency to rapidly return to straight position because of the characteristics that may explain more flexibility of this rotary system. According to the manufacturer of the Neoniti system, the use of a developed wire-cut electrical discharge machining (EDM) along with an appropriate heat treatment is responsible for this exceptional straightening resistance and capacity to preserve the original shape of the root canals, after preparation [20].

The canal preparation with Wave One files in a study in 2014 showed less straightening or transportation and better centering ability than One Shape (Micro-Mega) and ProTaper [21].

Burklein et al. reported less straightening or canal transportation for the reciproc(VDW) single file system than One Shape(Micro-Mega) although this difference was not statistically significant [22].

D^,^ Amario et al. also, in another comparative study between instrumentation with One Shape (Micro-Mega), Reciproc (VDW), and Wave One (Dentsply Maillefer) reciprocating systems, reported no statistically significant difference regarding canal transportation, faster preparation time among 3 experimental groups, and no instrument from any group separated in their study; all systems maintained the original canal shape well and were quick and safe to use [23].

Another study by Santa Rosa et al. on Wave One (Dentsply Maillefer) and One Shape (Micro-Mega) systems showed similar shaping ability in severely curved MB canals of maxillary molars, and both systems maintained the original canal anatomy of root canals well [24]. More straightening or canal transportation by the One Shape system in this study is consistent with most studies in the past which have reported more canal transportation by One Shape in comparison with other NiTi rotary systems [21–24]. A triangular or modified triangular cross section and three sharp cutting edges in the middle and apical thirds and an S-shaped cross section with two cutting edges near the shaft improve the cutting efficiency and, as a result, the speed of preparation in the One Shape system whilst decreasing flexibility in comparison with the Neoniti and other NiTi rotary systems that do not follow this structural design.

Using the same file for 5 times may cause a deteriorating effect on its instrumentation. This limitation existed for all files in both groups, although having no separated instrument in both groups shows that both systems have high fatigue strength. Regarding the effect of this deteriorating effect on the shaping ability and speed of preparation, this limitation impacts on the instruments in both groups similarly with low possibility of an overall influence on the averages of amounts calculated.

One Shape (Micro-Mega) is a single file and also a single-use or single-patient instrument, quick, safe, and with a simple or an easy-to-learn protocol that might be a good alternative for reciprocating single-file systems without the need to provide a special Endo motor with reciprocation motion, so both Neoniti and One Shape single-file systems can be used with simple continuous motion [25].

## 5. Conclusions

Based on the results and within the limitations of this study, the Neoniti rotary system maintains the original shape of curved root canals better than the One Shape. Although according to the statistical analysis, preparation time in the One Shape group was significantly less, in clinical practice, this small difference could be overlooked; also, in clinical practice, better technical performance may be more important than speed.

## Figures and Tables

**Table 1:** Amount of dentin removed from mesial and distal walls of MB canals.

Section	File	Mean (SD)	95% CI		Min	Max	*P* value
Mesial 2 mm	One Shape	0.0715 (0.01496)	0.0645	0.0785	0.05	0.09	0.0001
	Neoniti	0.0100 (0.00795)	0.0063	0.0137	0.0	0.02	

Mesial 4 mm	One Shape	0.0630 (0.01380)	0.0565	0.0695	0.04	0.08	0.0001
	Neoniti	0.0220 (0.0110)	0.0168	0.0272	0.01	0.04	

Mesial 6 mm	One Shape	0.0520 (0.0115)	0.0466	0.0574	0.03	0.07	0.005
	Neoniti	0.0410 (0.0121)	0.0353	0.0467	0.02	0.06	

Mesial 8 mm	One Shape	0.0450 (0.0119)	0.0394	0.0506	0.02	0.06	0.003
	Neoniti	0.0320 (0.0136)	0.0256	0.0384	0.01	0.05	

Distal 2 mm	One Shape	0.1640 (0.01903)	0.1551	0.1729	0.13	0.19	0.0001
	Neoniti	0.0220 (0.00951)	0.0175	0.0265	0.01	0.04	

Distal 4 mm	One Shape	0.1445 (0.01820)	0.1360	0.1530	0.11	0.17	0.0001
	Neoniti	0.0555 (0.01317)	0.0493	0.0617	0.03	0.08	

Distal 6 mm	One Shape	0.1220 (0.01963)	0.1128	0.1312	0.09	0.15	0.0001
	Neoniti	0.0955 (0.01395)	0.0890	0.1020	0.08	0.13	

Distal 8 mm	One Shape	0.0920 (0.01152)	0.0866	0.0974	0.07	0.11	0.0001
	Neoniti	0.0715 (0.01631)	0.0639	0.0791	0.0.4	0.1	

**Table 2:** Amount of canal transportation.

Section (mm)	File	Mean (SD)	95% CI		Min	Max	*P* value
2	Neoniti	0.0130 (0.00865)	0.0090	0.0170	0.0	0.03	0.0001
	One Shape	0.0955 (0.02038)	0.0860	0.1050	0.05	0.13	

4	Neoniti	0.0315 (0.00988)	0.0269	0.0361	0.02	0.05	0.0001
	One Shape	0.0820 (0.02142)	0.0720	0.0920	0.04	0.12	

6	Neoniti	0.0545 (0.01099)	0.0494	0.0596	0.04	0.07	0.0001
	One Shape	0.0815 (0.02159)	0.0714	0.0916	0.03	0.11	

8	Neoniti	0.0445 (0.01099)	0.0394	0.0496	0.03	0.06	0.008
	One Shape	0.0575 (0.01743)	0.0493	0.0657	0.02	0.09	

**Table 3:** Preparation time (in seconds) for rotary systems.

Group	N	Mean (SD)	Minimum	Maximum	95% CI		*P* value
One Shape	20	138.138 (2.662)	132.91	142.03	−8.658	−4.674	0.0001
Neoniti	20	144.804 (3.503)	138.07	150.00	−8.663	−4.669	

## Data Availability

Data for this study are available upon request.
